# Empowering Clinical Decision Making in Oligometastatic Colorectal Cancer: The Potential Role of Drug Screening of Patient-Derived Organoids

**DOI:** 10.1200/PO.21.00143

**Published:** 2021-07-21

**Authors:** Gianluca Mauri, Erika Durinikova, Alessio Amatu, Federica Tosi, Andrea Cassingena, Francesco Rizzetto, Kristi Buzo, Pamela Arcella, Maria Costanza Aquilano, Emanuela Bonoldi, Silvia Marsoni, Salvatore Siena, Alberto Bardelli, Andrea Sartore-Bianchi, Sabrina Arena

**Affiliations:** ^1^ Niguarda Cancer Center, Grande Ospedale Metropolitano Niguarda, Milan, Italy; ^2^ Department of Oncology and Hemato-Oncology, Università degli Studi di Milano, Milan, Italy; ^3^ IFOM-FIRC Institute of Molecular Oncology, Milan, Italy; ^4^ Candiolo Cancer Institute, FPO—IRCCS, Candiolo, Torino, Italy; ^5^ Department of Oncology, University of Torino, Candiolo, Torino, Italy

## INTRODUCTION

The definition of oligometastatic colorectal cancer (CRC) identifies a peculiar subpopulation of patients characterized by a limited metastatic spread of disease.^1^ Oligometastatic disease is defined as the involvement of up to two or occasionally three sites with five or sometimes more metastases that for their anatomic localization is amenable to local ablative therapies, thus rendering the patient free of disease.^1-3^ Thus, this subgroup is wide and has a significant cohort of patients with CRC. Among patients with oligometastatic CRC, those with liver-limited disease represent a more refined subset and should always be discussed in multidisciplinary teams since they appear to more likely benefit from multimodal approaches with curative intent.^2,4-6^ A perioperative systemic treatment integrated with surgical liver metastasectomy should be regarded as the best multimodal approach.^2,7^ However, the best drug regimen to be adopted for this subset of patients with CRC is still debatable and should be tailored case-by-case.

Considering left-sided microsatellite stable, *RAS* and *BRAF* wild-type CRC, doublet cytotoxic regimens (FOLFIRI or FOLFOX) plus an anti-epidermal growth factor receptor (EGFR) drug can represent the best option on the basis of significant response rate (RR).^8-10^ However, triplet cytotoxic regimens FOLFOXIRI plus bevacizumab or anti-EGFR drug demonstrate an impressive RR up to 87%, which are increasingly regarded as potential novel neoadjuvant standard strategies.^5,11^ However, severe treatment-related toxicities have been reported in up to 80% of patients.^11,12^ Hence, both doublet or triplet combinations and anti-EGFR or antivascular endothelial growth factor are feasible options in this subset of patients.

The generation of preclinical models such as patient-derived organoids (PDOs), recapitulating patient tumor histology and genetics, is emerging as a tool to predict treatment efficacy in oncology.^13,14^ Although genomics has already improved treatment choice in patients with CRC, especially for those carrying *RAS*/*BRAF* wild type, coclinical trials are becoming more and more important to directly test different treatment options in patient-derived tumors.^15^

Here, we present a proof-of-concept case report about the potential role of drug sensitivity testing in PDOs in the clinical decision making of oligometastatic CRC.

## TRANSLATIONAL REPORT

**FIG 1.**
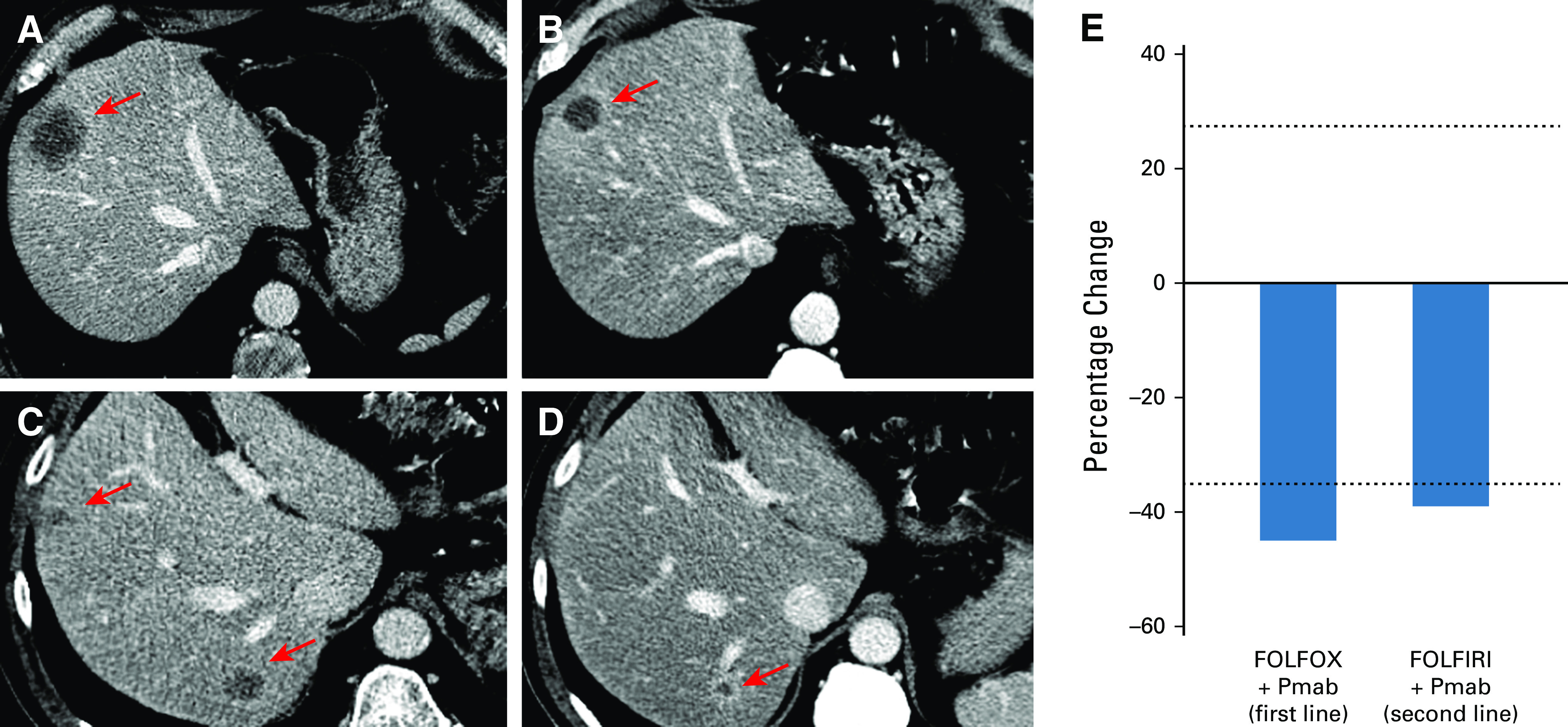
Radiologic evidence of response following FOLFOX plus panitumumab and FOLFIRI plus panitumumab. (A) The venous phase of the baseline CT scan performed in July 2018 showed a large hypodense lesion in (arrow) segment VIII of the liver. (B) Following four cycles of treatment with FOLFOX and panitumumab, at CT scan performed on November 2018, a partial response (shrinkage of the longest lesion's diameter from 38 to 20 mm) of the hepatic lesion in (arrow) segment VIII was observed as well. (C) The venous phase of the CT scan performed in October 2019 demonstrated liver relapse of disease with three new hypodense lesions with peripheral rim enhancement in segment VII, segments V-VIII, and (not visible) segment V. (D) After six cycles of FOLFIRI and panitumumab, the CT scan performed in January 2020 showed a decrease in lesions' size: segment V decrease by 50% (from 12 to 6 mm), segments V-VIII by 37% (from 19 to 12 mm), and segment VII by 33% (from 18 to 12 mm). All lesions were evaluated as per RECIST1.1 criteria. (E) Bar graph summarizes the best RECIST1.1 response to both first-line treatment with FOLFOX plus panitumumab (–45%) and second-line treatment with FOLFIRI plus panitumumab (–39%). CT, computed tomography; Pmab, panitumumab.

**FIG 2.**
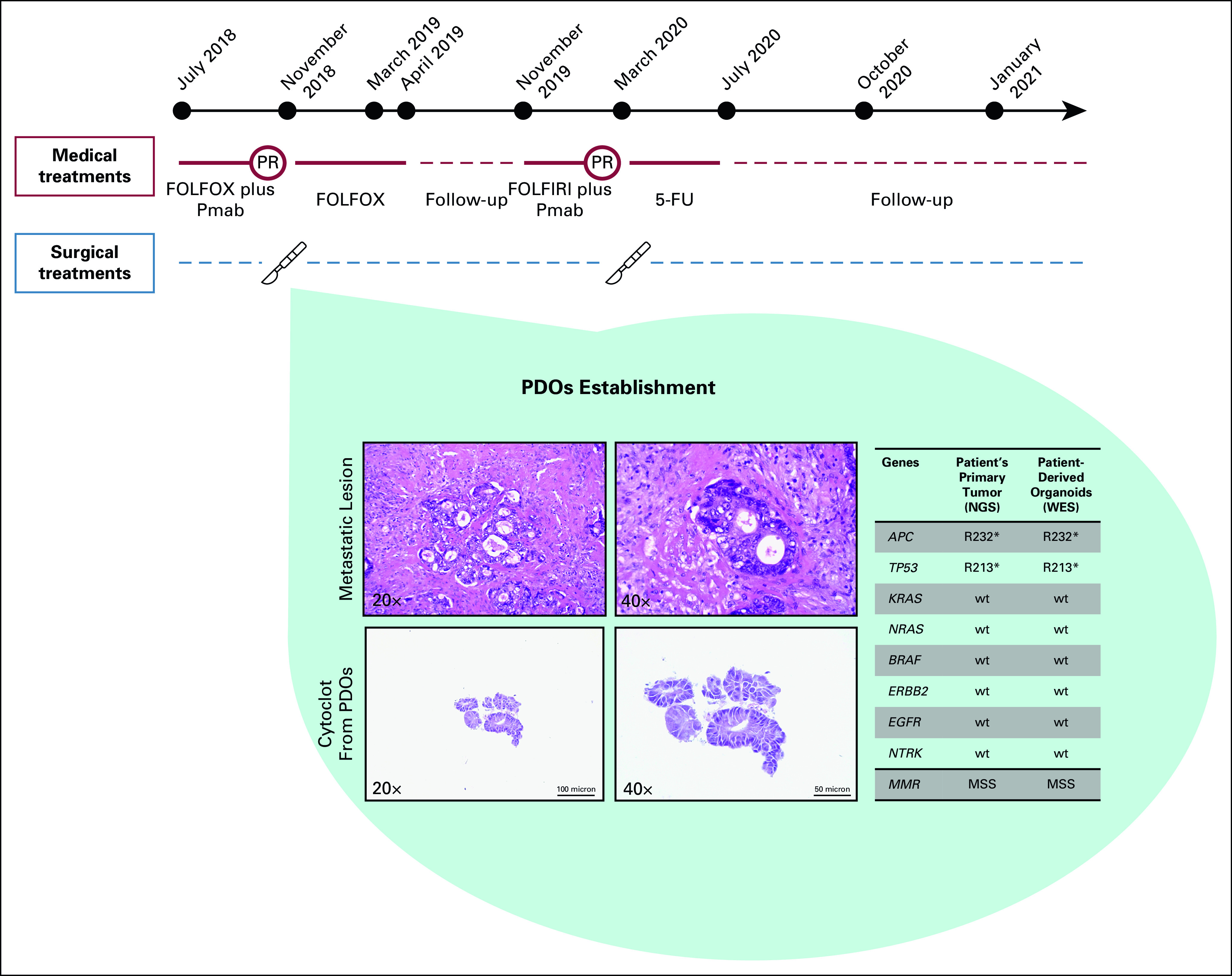
Patient's oncologic history timeline and histopathologic analysis of the metastatic lesion and of the PDOs. In the light blue background, representative hematoxylin and eosin images from the metastatic lesion recovered in March 2018 (top panels) and from the organoids derived from this lesion (lower panels) are given. Furthermore, the table in the light blue background depicts molecular alterations found in genes that are known to play a key role in colorectal cancer development and are recommended by clinical guidelines to define patients' treatment. Data were obtained from the NGS panel (FoundationOne CDx) performed according to GO40782 clinical trial (NCT02568267) screening on archival patient's left-sided primary tumor tissue and from the WES performed on PDOs. 5-FU, fluorouracil; Lancet, surgical procedure; MMR, mismatch repair status; MSS, microsatellite stable; NGS, next-generation sequencing; PDOs, patient-derived organoids; Pmab, panitumumab; PR, partial response; red circle, best response at computed tomography scan disease reassessment; WES, whole-exome sequencing.

In July 2018, a 56-year-old healthy man underwent a screening colonoscopy and was diagnosed with a left-sided colonic mass demonstrating a moderately differentiated (G2) adenocarcinoma. Afterward, a computed tomography (CT) scan revealed two metastatic lesions in liver segments VIII and V (Fig 1A). Baseline carcinoembryonic antigen (CEA) was 15.5 ng/mL, and cancer antigen (CA) 19.9 was 55 U/mL. Following multidisciplinary discussion and on the basis of standard molecular biomarker assessment, demonstrating microsatellite stability, no mutations in *RAS* and *BRAF*, and no *ERBB2* amplification, the patient received four cycles of neoadjuvant FOLFOX plus panitumumab (Fig 2). Treatment was complicated by grade 3 afebrile neutropenia requiring peg-filgrastim support. No other adverse events occurred.

CT scan reassessment demonstrated partial response (PR) (Figs 1B and 1E), and thus, following a new multidisciplinary discussion, in November 2018, the patient underwent R0 liver metastasectomy and left emicolectomy. The pathology report described an ypT3 ypN2b ypM1aG2 adenocarcinoma. At this time, after signing informed consent and the enrollment in the AlfaOmega observational trial (NCT04120935), a fresh tumor sample from the metastatic liver lesion in segment VIII was used for PDOs generation. Once established (Data Supplement), these tumoroids underwent histologic analysis, confirming the correspondence and similarity between the PDOs and the metastatic tissue of origin (Fig 2). The next-generation sequencing performed by the FoundationOne CDx panel on primary left colon lesion, as per GO40782 clinical trial screening (NCT02568267), revealed no mutations discriminating for sensitivity to cytotoxic agents.^2,6,16^ We also performed whole-exome sequencing analysis on PDOs, confirming the presence of patient-specific trunk alterations, whereas no alterations conferring resistance to anti-EGFR drugs were observed (Fig 2).

Following surgical resection, as per clinical practice, the patient received further eight cycles of systemic treatment with FOLFOX regimen supported with peg-filgrastim prophylaxis, ending in April 2019. The patient tolerated the postoperative treatment well without relevant side effects.

**FIG 3.**
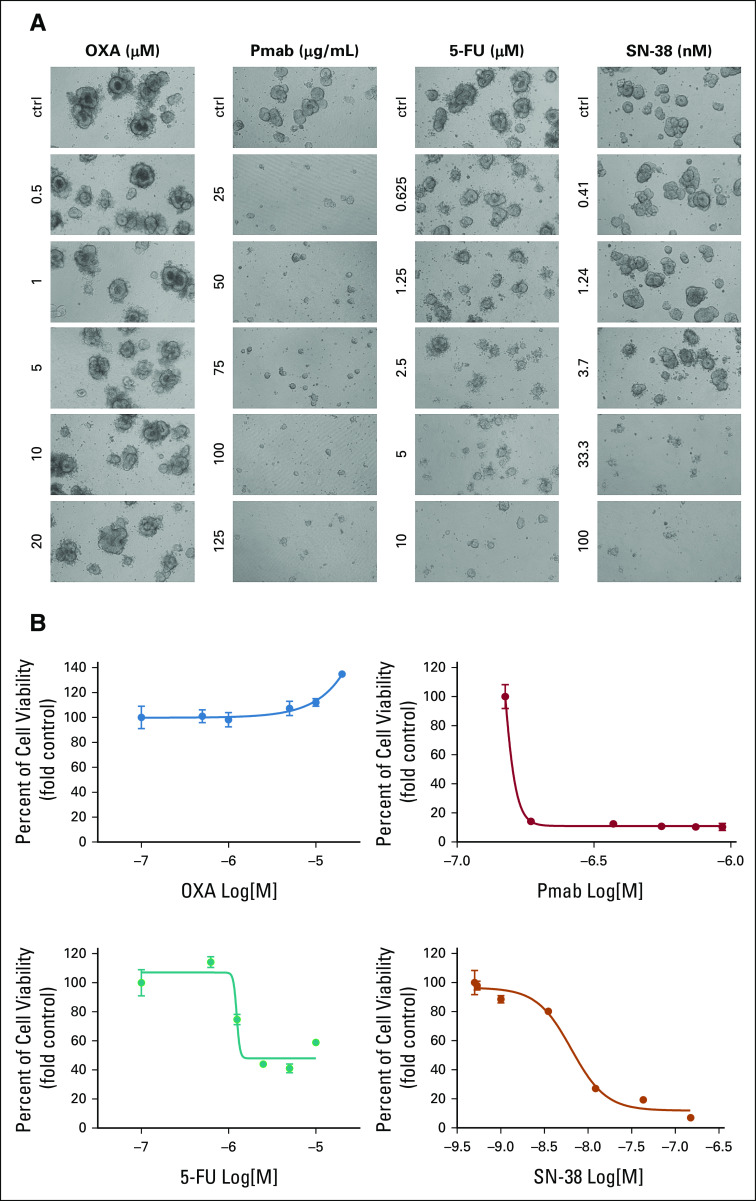
Patient-derived organoids show different profiles of sensitivity to cytotoxic drugs and targeted treatment. (A) Representative bright-field microscopy images of organoids derived from the oligometastatic patient treated with OXA and 5-FU for 12 days and with SN-38 and panitumumab for 7 days. MG-132 was used as a positive control for organoids' death (data not shown), and DMSO or DMEM/F12 served as a negative control. Magnification: 10×. (B) At the end of the treatment, organoids' viability was measured by the CellTiter GLO assay and numbers were analyzed using GraphPad software. The results are the average of at least two independent experiments with technical quadruplicates. Error bars represent the standard deviation among biologic replicates. Z factor values varied between 0.73 and 0.91. ctrl, control; 5-FU, fluorouracil; OXA, oxaliplatin; Pmab, panitumumab.

**FIG A1.**
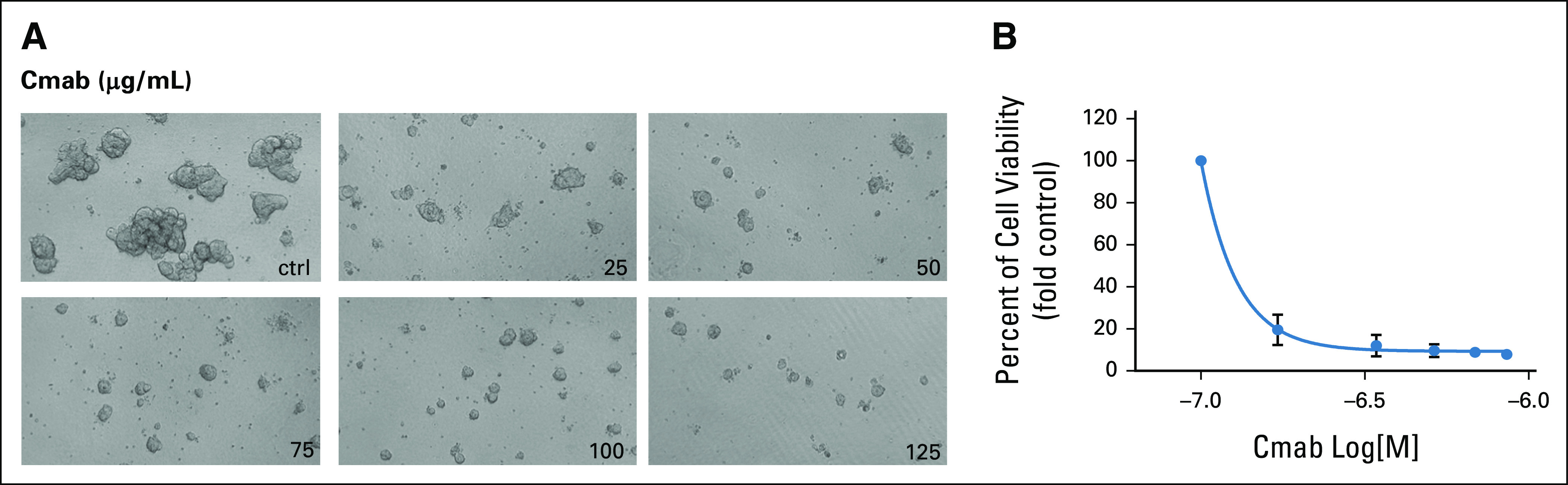
The response of patient-derived organoids to the anti-EGFR monoclonal antibody cetuximab. (A) Representative bright-field microscopy images of organoids treated with cetuximab for 7 days. MG-132 was used as a positive control for organoids' death (data not shown), whereas DMEM/F12 medium served as a negative control. Magnification: 10×. (B) At the end of the treatment, organoids' viability was measured by the CellTiter GLO assay and viability values were analyzed using GraphPad software. The results are the average of three independent experiments with technical quadruplicates, and error bars represent the standard deviation among biologic replicates. Z factor values varied between 0.75 and 0.82. Cmab, cetuximab; ctrl, control.

Despite this multimodal approach, CT and positron emission tomography scan in October 2019 showed disease relapse in liver segments VIII, VII, and V (Fig 1C). Considering this evidence, the case was discussed collectively and we also took into consideration the results of a drug sensitivity testing performed on the PDOs, which, evaluating the most effective agents in this clinical context, showed complete resistance to oxaliplatin (OXA) but rather a prominent sensitivity to fluorouracil 5-FU SN-38 (the active metabolite of irinotecan), panitumumab, and cetuximab (Fig 3 and Appendix Fig A1). Primarily based on clinical standard and corroborated by PDOs drug sensitivity testing, we avoided OXA-based treatment and the patient was treated with six cycles of FOLFIRI and panitumumab and peg-filgrastim prophylaxis. This schedule was well-tolerated, and the following CT scan again demonstrated a PR (Figs 1D and 1E). Given this result, in March 2020, the patient underwent multiple liver metastasectomy confirming metastatic adenocarcinoma. Thereafter, the patient received six cycles of postoperative 5-FU, again well-tolerated. In July 2020, the CT scan demonstrated no evidence of disease and CEA and CA19.9 were normal. The following CT scan in October 2020 confirmed no relapse with normal CEA and CA19.9. The patient has consented to the submission and the publication of the case report.

## DISCUSSION

Our groups have jointly focused their efforts on the development of preclinical models with the aim of tailoring treatment for patients with metastatic CRC on the basis of the results obtained from the pharmacologic screening on patients' avatars.^17-24^ By taking advantage of patient-derived xenografts,^19^ we previously identified optimal therapeutic regimens for CRC druggable targets such as *ERBB2* amplification.^25-27^ Recently, the establishment of organoids directly obtained from surgical procedure or tumor biopsy has accelerated the possibility to test drugs on patients' avatars,^21,23,28^ thus offering a valid approach to test drug sensitivity on the bench, in parallel to patient care.

Here, we report the case of an patient with oligometastatic CRC whose drug screening on PDOs resembles both resistance and sensitivity to main cytotoxic agents. Most notably, PDOs drug testing was performed in parallel with clinical care. This translational paralleling is usually hampered by the time required to establish PDOs and test drug activity in the continuum of care of CRC (Fig 2). However, the expected recurrence-free survival following liver resection in oligometastatic CRC offers a timely opportunity to translate the PDOs drug sensitivity testing results into clinical decision making at relapse. Here, we were able to identify a medical perioperative regimen with the higher chance to obtain tumor shrinkage, aiming to offer a second potentially curative resection to the patient.^2,7^ Indeed, given performance status, tumor sidedness, and molecular status, both a triple cytotoxic regimen composed of FOLFOXIRI plus bevacizumab and a doublet cytotoxic combination plus anti-EGFR drug were feasible, reasonably expecting similar RR.^2,12^ However, PDO's pharmacogenomics profile showing resistance to OXA and sensitivity to 5-FU, SN-38, and panitumumab supported the clinical choice of FOLFIRI and panitumumab as perioperative regimen. This led to remarkable disease shrinkage, allowing for a second liver metastasectomy to improve the chance of cure, sparing side effects of other intensive cytotoxic regimens.^11,29-31^ Here, we limited our screening to approved drugs, but elsewhere, we already proposed rationale combinations including unapproved therapeutics on the basis of the results from drug screening on PDOs.^17,32,33^

This case report reinforces other recent publications in which PDOs were suggested as a potential platform to identify the best treatment for each patient.^17^ In addition, we envision the subset of patients with oligometastatic CRC as the optimal to bypass the limitation given by time required to PDOs establishment. Prospective translational trials are needed to verify the feasibility of this translational approach in peculiar clinical settings (Table 1).

**TABLE 1.**
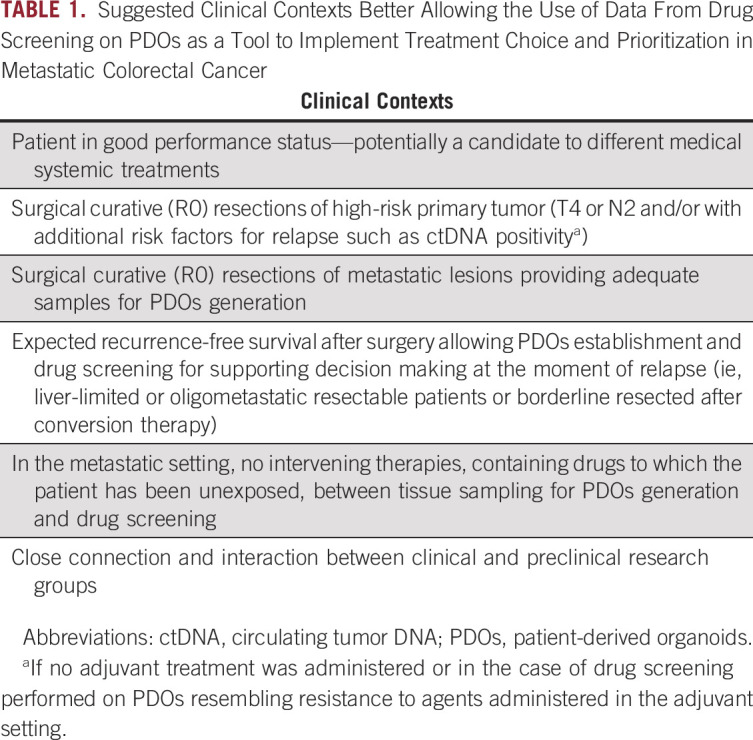
Suggested Clinical Contexts Better Allowing the Use of Data From Drug Screening on PDOs as a Tool to Implement Treatment Choice and Prioritization in Metastatic Colorectal Cancer

Treatment implementation driven by PDOs drug sensitivity testing has intrinsic limitations potentially hampering its applicability in the daily clinical practice. First, limiting factors such as tumor heterogeneity leading to potential sampling biases might represent relevant barriers toward translational applicability. Thus, we hypothesize that PDOs testing might be more helpful in patients with a low tumor burden, because of intrapatient heterogeneity that is expected to be lower in these patients.^34,35^ Indeed, diffused metastatic disease is more likely to be widely heterogeneous and patients do not achieve the same results observed on PDOs.^21,36,37^ Second, PDOs drug sensitivity testing constitutes a relatively expensive method to choose a systemic cytotoxic regimen. However, we reasoned that this might be justifiable to maximize the chance of cure and improve the quality of life. Finally, in hospitals where a close connection with a preclinical laboratory is not a reality, PDO-driven approach might be difficult to be set up.

In conclusion, we provide a translational report demonstrating that PDOs drug sensitivity testing is feasible and might represent a valid tool to predict and improve the choice for the best perioperative personalized approach in patients with oligometastatic CRC. We suggest indeed that this setting, because of the time intervals between surgery and resumption of medical treatment, would offer the best choice to parallel patients' care, allowing timely modulation of the treatment strategy. These advantages should be supported by optimization of protocol standardization and balance against costs related to organoid maintenance.

## References

[1] Weichselbaum RR Hellman S Oligometastases revisited Nat Rev Clin Oncol 8 378–382 2011 21423255 10.1038/nrclinonc.2011.44

[2] Van Cutsem E Cervantes A Adam R et al ESMO consensus guidelines for the management of patients with metastatic colorectal cancer Ann Oncol 27 1386–1422 2016 27380959 10.1093/annonc/mdw235

[3] Hellman S Weichselbaum RR Oligometastases J Clin Oncol 13 8–10 1995 7799047 10.1200/JCO.1995.13.1.8

[4] Ruers T, Van Coevorden F, Punt CJA (2017). Local treatment of unresectable colorectal liver metastases: Results of a randomized phase II trial. J Natl Cancer Inst.

[5] Moretto R Rossini D Zucchelli G et al Oligometastatic colorectal cancer: Prognosis, role of locoregional treatments and impact of first-line chemotherapy-a pooled analysis of TRIBE and TRIBE2 studies by Gruppo Oncologico del Nord Ovest Eur J Cancer 139 81–89 2020 32979645 10.1016/j.ejca.2020.08.009

[6] Benson AB Venook AP Al-Hawary MM et al Colon cancer, version 2.2021, NCCN clinical practice guidelines in oncology J Natl Compr Cancer Netw 19 329–359 2021 10.6004/jnccn.2021.0012 33724754

[7] Adam R De Gramont A Figueras J et al The oncosurgery approach to managing liver metastases from colorectal cancer: A multidisciplinary international consensus Oncologist 17 1225–1239 2012 22962059 10.1634/theoncologist.2012-0121 PMC3481888

[8] Heinemann V von Weikersthal LF Decker T et al FOLFIRI plus cetuximab versus FOLFIRI plus bevacizumab as first-line treatment for patients with metastatic colorectal cancer (FIRE-3): A randomised, open-label, phase 3 trial Lancet Oncol 15 1065–1075 2014 25088940 10.1016/S1470-2045(14)70330-4

[9] Douillard J-Y Oliner KS Siena S et al Panitumumab-FOLFOX4 treatment and RAS mutations in colorectal cancer N Engl J Med 369 1023–1034 2013 24024839 10.1056/NEJMoa1305275

[10] Boeckx N Koukakis R Op de Beeck K et al Primary tumor sidedness has an impact on prognosis and treatment outcome in metastatic colorectal cancer: Results from two randomized first-line panitumumab studies Ann Oncol 28 1862–1868 2017 28449055 10.1093/annonc/mdx119 PMC5834073

[11] Modest DP Martens UM Riera-Knorrenschild J et al FOLFOXIRI plus panitumumab as first-line treatment of RAS wild-type metastatic colorectal cancer: The randomized, open-label, phase II VOLFI study (AIO KRK0109) J Clin Oncol 37 3401–3411 2019 31609637 10.1200/JCO.19.01340

[12] Cremolini C, Antoniotti C, Stein A (2020). Individual patient data meta-analysis of FOLFOXIRI plus bevacizumab versus doublets plus bevacizumab as initial therapy of unresectable metastatic colorectal cancer. J Clin Oncol.

[13] Tuveson D Clevers H Cancer modeling meets human organoid technology Science 364 952–955 2019 31171691 10.1126/science.aaw6985

[14] Nagle PW Plukker JTM Muijs CT et al Patient-derived tumor organoids for prediction of cancer treatment response Semin Cancer Biol 53 258–264 2018 29966678 10.1016/j.semcancer.2018.06.005

[15] Hedayat S Valeri N Patient-derived organoids: Promises, hurdles and potential clinical applications Clin Oncol (R Coll Radiol) 32 213–216 2020 31926819 10.1016/j.clon.2019.12.009

[16] Yoshino T Arnold D Taniguchi H et al Pan-Asian adapted ESMO consensus guidelines for the management of patients with metastatic colorectal cancer: A JSMO-ESMO initiative endorsed by CSCO, KACO, MOS, SSO and TOS Ann Oncol 29 44–70 2018 29155929 10.1093/annonc/mdx738

[17] Arena S Corti G Durinikova E et al A subset of colorectal cancers with cross-sensitivity to olaparib and oxaliplatin Clin Cancer Res 26 1372–1384 2020 31831554 10.1158/1078-0432.CCR-19-2409

[18] Lazzari L Corti G Picco G et al Patient-derived xenografts and matched cell lines identify pharmacogenomic vulnerabilities in colorectal cancer Clin Cancer Res 25 6243–6259 2019 31375513 10.1158/1078-0432.CCR-18-3440 PMC7611232

[19] Bertotti A Migliardi G Galimi F et al A molecularly annotated platform of patient-derived xenografts (“xenopatients”) identifies HER2 as an effective therapeutic target in cetuximab-resistant colorectal cancer Cancer Discov 1 508–523 2011 22586653 10.1158/2159-8290.CD-11-0109

[20] Bertotti A Papp E Jones S et al The genomic landscape of response to EGFR blockade in colorectal cancer Nature 526 263–267 2015 26416732 10.1038/nature14969 PMC4878148

[21] Ooft SN, Weeber F, Dijkstra KK (2019). Patient-derived organoids can predict response to chemotherapy in metastatic colorectal cancer patients. Sci Transl Med.

[22] Hidalgo M Amant F Biankin AV et al Patient-derived xenograft models: An emerging platform for translational cancer research Cancer Discov 4 998–1013 2014 25185190 10.1158/2159-8290.CD-14-0001 PMC4167608

[23] Vlachogiannis G Hedayat S Vatsiou A et al Patient-derived organoids model treatment response of metastatic gastrointestinal cancers Science 359 920–926 2018 29472484 10.1126/science.aao2774 PMC6112415

[24] Durinikova E, Buzo K, Arena S (2021). Preclinical models as patients' avatars for precision medicine in colorectal cancer: Past and future challenges. J Exp Clin Cancer Res.

[25] Sartore-Bianchi A Trusolino L Martino C et al Dual-targeted therapy with trastuzumab and lapatinib in treatment-refractory, KRAS codon 12/13 wild-type, HER2-positive metastatic colorectal cancer (HERACLES): A proof-of-concept, multicentre, open-label, phase 2 trial Lancet Oncol 17 738–746 2016 27108243 10.1016/S1470-2045(16)00150-9

[26] Tosi F Sartore-Bianchi A Lonardi S et al Long-term clinical outcome of trastuzumab and lapatinib for HER2-positive metastatic colorectal cancer Clin Colorectal Cancer 19 256–262.e2 2020 32919890 10.1016/j.clcc.2020.06.009

[27] Sartore-Bianchi A, Lonardi S, Martino C (2020). Pertuzumab and trastuzumab emtansine in patients with HER2-amplified metastatic colorectal cancer: The phase II HERACLES-B trial. ESMO Open.

[28] Yao Y Xu X Yang L et al Patient-derived organoids predict chemoradiation responses of locally advanced rectal cancer Cell Stem Cell 26 17–26.e6 2020 31761724 10.1016/j.stem.2019.10.010

[29] Mauri G Bencardino K Sartore-Bianchi A et al Toxicity of oxaliplatin rechallenge in metastatic colorectal cancer Ann Oncol 29 2143–2144 2018 10.1093/annonc/mdy306 30060112

[30] Mauri G, Gori V, Bonazzina E (2020). Oxaliplatin retreatment in metastatic colorectal cancer: Systematic review and future research opportunities. Cancer Treat Rev.

[31] Bencardino K Mauri G Amatu A et al Oxaliplatin immune-induced syndrome occurs with cumulative administration and rechallenge: Single institution series and systematic review study Clin Colorectal Cancer 15 213–221 2016 26979913 10.1016/j.clcc.2016.02.001

[32] Lorenzato A, Magrì A, Matafora V (2020). Vitamin C restricts the emergence of acquired resistance to EGFR-targeted therapies in colorectal cancer. Cancers (Basels).

[33] Amodio V Yaeger R Arcella P et al EGFR blockade reverts resistance to KRASG12C inhibition in colorectal cancer Cancer Discov 10 1129–1139 2020 32430388 10.1158/2159-8290.CD-20-0187 PMC7416460

[34] Caswell DR, Swanton C (2017). The role of tumour heterogeneity and clonal cooperativity in metastasis, immune evasion and clinical outcome. BMC Med.

[35] Siravegna G Lazzari L Crisafulli G et al Radiologic and genomic evolution of individual metastases during HER2 blockade in colorectal cancer Cancer Cell 34 148–162.e7 2018 29990497 10.1016/j.ccell.2018.06.004

[36] Parikh AR Leshchiner I Elagina L et al Liquid versus tissue biopsy for detecting acquired resistance and tumor heterogeneity in gastrointestinal cancers Nat Med 25 1415–1421 2019 31501609 10.1038/s41591-019-0561-9 PMC6741444

[37] McGranahan N Swanton C Biological and therapeutic impact of intratumor heterogeneity in cancer evolution Cancer Cell 27 15–26 2015 25584892 10.1016/j.ccell.2014.12.001

